# Importin-Mediated Pathological Tau Nuclear Translocation Causes Disruption of the Nuclear Lamina, TDP-43 Mislocalization and Cell Death

**DOI:** 10.3389/fnmol.2022.888420

**Published:** 2022-05-03

**Authors:** Robert F. Candia, Leah S. Cohen, Viktoriya Morozova, Christopher Corbo, Alejandra D. Alonso

**Affiliations:** ^1^Department of Biology, Center for Developmental Neuroscience, College of Staten Island, The City University of New York, Staten Island, NY, United States; ^2^Biology Program, The Graduate Center, The City University of New York, New York, NY, United States; ^3^Department of Chemistry, College of Staten Island, The City University of New York, Staten Island, NY, United States; ^4^Department of Biological Sciences, Wagner College, Staten Island, NY, United States

**Keywords:** tau, nucleocytoplasmic transport, neurodegeneration, importin, Alzheimer’s disease

## Abstract

Tau is a cytosolic protein that has also been observed in the nucleus, where it has multiple proposed functions that are regulated by phosphorylation. However, the mechanism underlying the nuclear import of tau is unclear, as is the contribution of nuclear tau to the pathology of tauopathies. We have previously generated a pathological form of tau, PH-tau (pseudophosphorylation mutants S199E, T212E, T231E, and S262E) that mimics AD pathological behavior in cells, *Drosophila*, and a mouse model. Here, we demonstrated that PH-tau translocates into the nucleus of transiently transfected HEK-293 cells, but wildtype tau does not. We identified a putative importin binding site in the tau sequence, and showed that disruption of this site prevents tau from entering the nucleus. We further showed that this nuclear translocation is prevented by inhibitors of both importin-α and importin-β. In addition, expression of PH-tau resulted in an enlarged population of dying cells, which is prevented by blocking its entry into the nucleus. PH-tau-expressing cells also exhibited disruption of the nuclear lamina and mislocalization of TDP-43 to the cytoplasm. We found that PH-tau does not bundle microtubules, and this effect is independent of nuclear translocation. These results demonstrate that tau translocates into the nucleus through the importin-α/β pathway, and that PH-tau exhibits toxicity after its nuclear translocation. We propose a model where hyperphosphorylated tau not only disrupts the microtubule network, but also translocates into the nucleus and interferes with cellular functions, such as nucleocytoplasmic transport, inducing mislocalization of proteins like TDP-43 and, ultimately, cell death.

## Introduction

Tauopathies are a family of neurodegenerative diseases linked by a shared mechanism that involves the accumulation of abnormal tau protein. This family includes a wide array of diseases, such as Alzheimer’s disease (AD), frontotemporal dementia with Parkinsonism linked to chromosome 17 (FTDP-17), and amyotrophic lateral sclerosis (ALS). While each disease has a different phenotype, the formation of tau inclusions that ultimately result in neurodegeneration is central to the pathology of every tauopathy, implicating abnormal tau as a driving force behind these various diseases. This was unequivocally demonstrated by the identification of mutations in the tau gene, MAPT, that are sufficient to cause the development of FTDP-17 (Hutton et al., 1998; Poorkaj et al., 1998). While not every tauopathy has associated causative MAPT mutations, it has been consistently found that tau pathology is strongly correlated with the degree of cognitive decline, solidifying its importance in the disease progression (Arriagada et al., 1992; Nelson et al., 2012; Malpetti et al., 2020).

The most well-known role for tau is as a microtubule-binding protein where, under normal conditions, phosphorylation of tau causes the protein to lose its affinity for microtubules in a reversible manner (Weingarten et al., 1975; Lindwall and Cole, 1984; Mandelkow et al., 1995). In tauopathies, tau becomes irreversibly hyperphosphorylated resulting in accumulation to form tauopathy-related lesions. Studies using tau isolated from AD brains (AD P-tau) have found that tau behaves as a prion, with the ability to bind to normal tau (Alonso et al., 1996) and other microtubule-associated proteins (Alonso et al., 1997), sequestering them from the microtubules. AD P-tau itself cannot bind to microtubules (Alonso et al., 1994), but will bind to endogenous tau and trigger a conformational change, leading to destabilization of the microtubule network (Alonso et al., 1996, 2006, 2010) and disruption of axonal transport (LaPointe et al., 2009; Kanaan et al., 2011).

The presence of tau in the nucleus has been consistently shown for several decades, but its functions are not fully understood. The longest-standing role for nuclear tau is in DNA protection. Tau was first shown to interact with DNA over 40 years ago (Corces et al., 1978) and has a higher affinity for DNA than it does for microtubules (Corces et al., 1980). Stress conditions trigger a translocation of tau into the nucleus, where it binds to DNA in a reversible manner (Sultan et al., 2011). Importantly, the interaction between tau and DNA is destabilized by tau phosphorylation (Lu et al., 2013; Camero et al., 2014; Qi et al., 2015). Aside from this role, tau has also been implicated in mRNA stability. In this mechanism, p53 and PARN deadenylase, which have been associated with mRNA 3’ processing (Cevher et al., 2010; Devany et al., 2013), form a complex with tau and the isomerase Pin1, which can then regulate mRNA processing under both normal and stress conditions (Baquero et al., 2019). Additionally, formation of this complex is disrupted by tau phosphorylation, suggesting a potential role for alterations in mRNA processing and the DNA damage response in neurodegeneration (Baquero et al., 2019). There has also been a growing correlation between nuclear tau and chromatin regulation. Broad changes in histone acetylation and chromatin structure have been associated with pathological tau in tauopathies (Klein et al., 2019). More specifically, expression of tau with the FTDP-17-causing mutation R406W in Drosophila was sufficient to cause heterochromatin loss, aberrant gene expression, and neurotoxicity (Frost et al., 2014). Furthermore, the organization of pericentromeric heterochromatin is disrupted both when tau is knocked out and in the brains of AD patients (Mansuroglu et al., 2016).

During the last decade, research surrounding nucleocytoplasmic transport (NCT) in neurodegenerative disease has boomed. The regulation of NCT is mediated by nuclear pore complexes (NPCs) anchored in the nuclear envelope. These NPCs contain nucleoporin proteins that interact with nuclear transporters, namely importins. Importins first bind to nuclear localization sequences (NLSs) on proteins, and then to RanGDP, after which they interact with nucleoporins to bring the cargo through the nuclear envelope. Once inside, RanGDP is exchanged with RanGTP to release the cargo. Nuclear mRNAs are exported through a different mechanism, but still depend on interactions with nucleoporins (Carmody and Wente, 2009). Proper functioning of NCT pathways enables the export of mRNAs for translation and the import of critical proteins like transcription factors and stress response factors. On the other hand, disruption of NCT would lead to mislocalization of proteins and could promote abnormal protein-protein interactions, which are central to tauopathies. One recent study has shown that abnormal tau is capable of disrupting nucleoporins, leading to a cytoplasmic localization of typically nuclear reporter proteins (Eftekharzadeh et al., 2018). Additionally, TDP-43, a protein that is known to form aggregates in ALS and AD, has been shown to act in a similar manner to tau, disrupting NPCs and sequestering proteins (Freibaum et al., 2015; Jovičić et al., 2015; Zhang et al., 2015). In all, there is mounting evidence that disrupted NCT is central to tauopathies.

The goals of this study were to determine the mechanism of tau transport to the nucleus and to assess whether nuclear phospho-tau contributed to tauopathy-like pathology. We found that PH-tau (pseudophosphorylation mutants S199E, T212E, T231E, and S262E), but not wildtype tau (WT-tau), is able to translocate into the nucleus. Additionally, using NLStradamus software (Nguyen Ba et al., 2009), we predicted the existence of an NLS between amino acids 141 and 178 of tau, including a classical importin-α binding site (KKAK, amino acids 140–143). Interruption of this sequence by site-directed mutagenesis indicates its necessity for nuclear localization of tau. Further, pharmacological studies demonstrate that the combined action of importin-α and importin-β is necessary for the nuclear import of tau, consistent with the structure of the NLS. Flow cytometry with propidium iodide indicates significant toxicity of PH-tau, and that this toxicity depends on its nuclear localization. We provide evidence that NCT is altered in PH-tau-expressing cells by demonstrating disruption of the nuclear lamina and mislocalization of TDP-43, both of which require PH-tau entry into the nucleus. We also find that PH-tau is unable to bundle microtubules, and that this occurs independently from its nuclear translocation, suggesting that nuclear pathological tau may drive tauopathy alongside the well-characterized cytoskeletal disruption.

## Materials and Methods

### Reagents

Lipofectamine 2000 (#11668019), ivermectin (#J62777.MS), and propidium iodide (#P1304MP) were purchased from Invitrogen. Endofectin Max (#EF014) was purchased from GeneCopoeia. Importazole (#40110510MG) was purchased from MilliporeSigma. All cell culture reagents were purchased from Gibco (see section “Cell Culture”).

### Antibodies

Primary antibodies against TDP-43 (rabbit monoclonal, clone ARC0492, #MA535273, Lot #WL3448491), RAN (rabbit polyclonal, #PA579913, Lot #WG3334631A), GAPDH (rabbit polyclonal, #TAB1001, Lot # WC324413), and Histone H3 (rabbit polyclonal, #PA522388, Lot # WG33322671) were purchased from Invitrogen. Primary antibodies against Musashi-1 (rabbit polyclonal, #27185-1-AP, Lot #00051144) and Lamin B1 (rabbit polyclonal, #12987-1-AP) were purchased from Proteintech. Anti-α-tubulin primary antibody was purchased from the Developmental Studies Hybridoma Bank (product AA4.3). Anti-tau antibody was a gift from Dr. Nicholas Kanaan at Michigan State University (originally created by Dr. Lester Binder at Northwestern University). The following secondary antibodies were purchased from Invitrogen: anti-rabbit AlexaFluor594 (donkey, #A21207, Lot# 1938375), anti-mouse AlexaFluor594 (donkey, #A21203, Lot# 1918277), anti-rabbit AlexaFluor680 (goat, #A32734, Lot #VI308536), and anti-mouse AlexaFluor800 (goat, #A32730, Lot #UC279294).

### Cell Culture

All experiments were conducted in the HEK-293 cell line (ATCC). Cultures were maintained in 25 cm^2^ flasks (Corning) in DMEM/F-12 (Gibco #11320033) containing 10% fetal bovine serum (Gibco #A3160402), 1% L-glutamine (Gibco #A2916801), 1% sodium pyruvate (Gibco #11360070), and 1% penicillin-streptomycin (Gibco #15140122) at 5% CO2. Cultures were passaged twice per week upon reaching 90% confluency. Approximately 24 h before the intended start of an experiment, cultures were passaged and seeded in a 24-well plate on glass coverslips at a density of approximately 70,000 cells per well.

### Vectors

Expression vectors for WT-tau, PH-tau, PH-tau-R406W, and tau-R406W were described previously (Alonso et al., 2010). Each vector was modified by site-directed mutagenesis to create the mutations K140A and K141A (tau-K140A,K141A).

### Transfection

**FIGURE 1:**
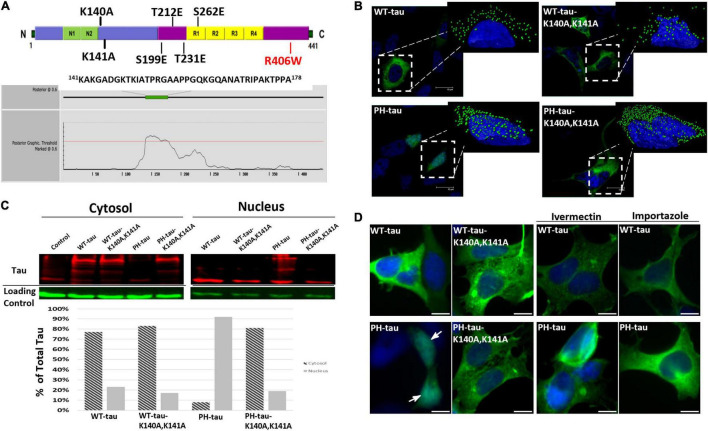
PH-tau translocates into the nucleus *via* the classical importin-α/importin-β pathway. **(A)** (Top) Diagram showing the locations of various mutations that were made to either simulate phosphorylation (S199E/T212E/T231E/S262E) or interrupt a putative nuclear localization signal (K140A/K141A). The R406W mutation is a common mutation found in frontotemporal dementia. (Bottom) Output from the NLStradamus prediction software, indicating its prediction of an NLS between amino acids 141 and 178. **(B)** HEK-293 cells were transfected with WT-tau, PH-tau, WT-tau-K140A,K141A, or PH-tau-K140A,K141A. Images are 63X magnification. (Insets) 3D reconstructions of individual tau-expressing cells were generated using IMARIS software to better visualize the nuclear localization of tau (green dots). Interruption of the putative NLS by mutation changes this localization. **(C)** Cytoplasmic and nuclear fraction from tau-transfected cells were separated by SDS-PAGE and stained for tau and loading controls (GAPDH for cytoplasm and Histone H3 for nucleus). These data confirm the microscopic visualization of PH-tau primarily in the nucleus and of PH-tau-K140A,K141A primarily in the cytoplasm. **(D)** Transfected cells were incubated with the importin-α inhibitor ivermectin, the importin-β inhibitor importazole, or in drug-free conditions. PH-tau, but not WT-tau, translocates into the nucleus (arrow). Inhibition of either importin is sufficient to prevent nuclear translocation of PH-tau. Scale bar indicates 15 μm.

**FIGURE 2:**
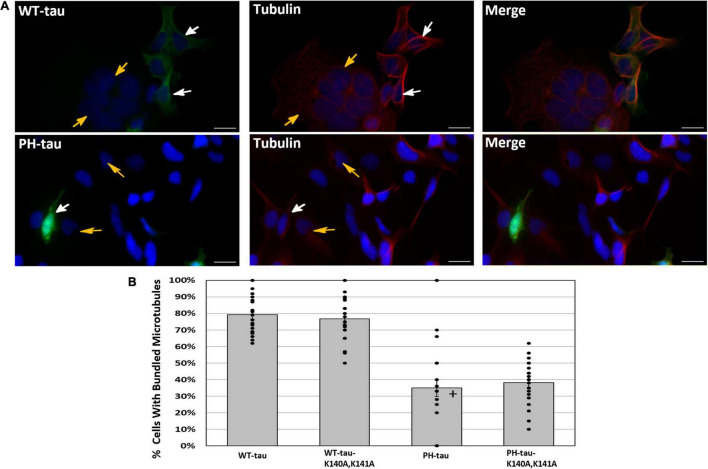
Microtubule bundling occurs in the presence of WT-tau but not PH-tau in a manner independent of its nuclear localization. **(A)** HEK-293 cells were transfected with WT-tau, PH-tau, WT-tau-K140A,K141A, or PH-tau-K140A,K141A and immunostained for α-tubulin. Microtubule bundling induced by WT-tau was determined by observation of a ring-like structure around the periphery of the cell. The top set shows the staining in WT-tau-transfected cells, and the bottom set of images shows the staining in PH-tau-transfected cells. The images demonstrate the presence of this structure in WT-tau-positive cells (white arrows, top), but not in tau-negative cells (gold arrows, top and bottom) or PH-tau-positive cells (white arrows, bottom). Scale bar indicates 30 μm. **(B)** The number of tau-positive cells in each group that displayed the indicated microtubule structure was counted and expressed as a percentage of all tau-positive cells. The mean percentage across all images (*n* = 20) was plotted as a bar graph. Error bars indicate SEM. The bars are overlayed with a dot plot of the percentages calculated from individual images (*n* = 20). Data were analyzed using Welch’s ANOVA, and Games-Howell *post-hoc* test was used for pairwise comparisons. A **+** indicates a significant difference from WT-tau (*p* = 0.013 vs. PH-tau; *p* = 0.001 vs. PH-tau-K140A,K141A) and from WT-tau-K140A,K141A (*p* = 0.001 vs. PH-tau; *p* = 0.031 vs. PH-tau-K140A,K141A).

**FIGURE 3:**
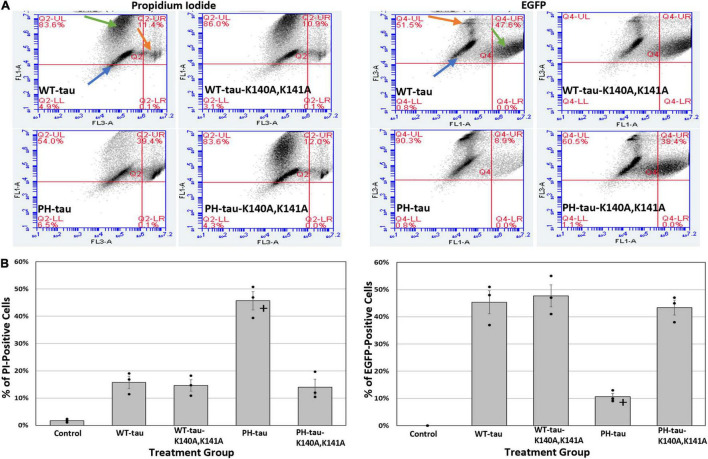
Translocation of PH-tau into the nucleus promotes cell death. **(A)** Graphs indicating the percentage of cells that are PI-positive (left set of four) or EGFP-positive (right set of four) 24 h after transfection with WT-tau, PH-tau, WT-tau-K140A,K141A, or PH-tau-K140A,K141A. Orange arrows indicate the population of PI-positive cells, green arrows indicate the population of EGFP-positive cells, and blue arrows indicate the population of cells that is neither EGFP-positive nor PI-positive. **(B)** The percentage of events (i.e., cells) was extracted from the upper-right quadrant of each graph and plotted. The left graph shows the percentage of cells that are PI-positive, while the right graph shows the percentage of cells that are EGFP-positive (i.e., tau positive). The average percentage across all 3 replicates of this experiment are plotted as bars, with error bars indicating SEM. The bars are overlayed with a dot plot showing the individual average percentages from each replicate (n = 3). Data were analyzed using Welch’s ANOVA, and Games-Howell *post-hoc* test was used for pairwise comparisons. The **+** indicates a significant difference in PH-tau from all other groups, where *p* < 0.001.

**FIGURE 4:**
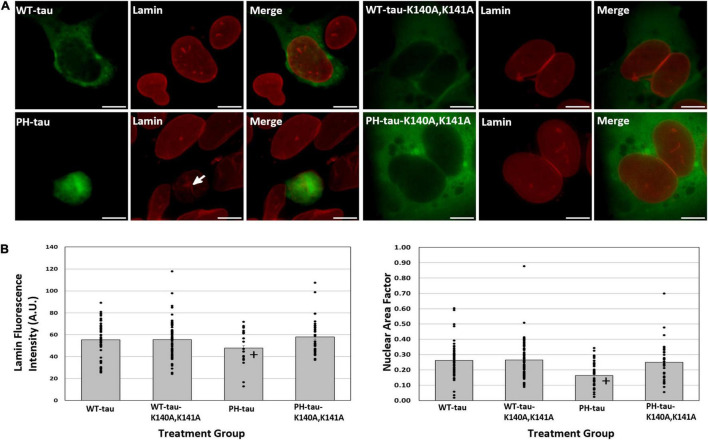
Nuclear PH-tau disrupts the nuclear lamina. **(A)** HEK-293 cells were transfected with WT-tau, PH-tau, WT-tau-K140A,K141A, or PH-tau-K140A,K141A (green) and then immunostained for lamin-B1 (red). The arrow indicates invagination of the nuclear membrane in a PH-tau-positive cell. Scale bar indicates 15 μm. **(B)** Left: The mean fluorescence intensity of lamin was quantified in individual tau-positive cells using ImageJ. The average fluorescence intensity across all cells (*n* = 49 for WT-tau, *n* = 75 for WT-tau-K140A,K141A, *n* = 38 for PH-tau, *n* = 37 for PH-tau-K140A,K141A) was plotted, with error bars showing SEM. The bars are overlayed with a dot plot showing the lamin fluorescence intensity for each cell. Data were analyzed using Welch’s ANOVA, and Games-Howell *post-hoc* test was used for pairwise comparisons The + indicates a significant difference between PH-tau and PH-tau-K140A,K141A (*p* = 0.008). No significance was detected in comparing WT-tau and PH-tau. Right: ImageJ was used to quantify the area and roundness of each tau-positive nucleus (*n* = 49 for WT-tau, *n* = 75 for WT-tau-K140A,K141A, *n* = 38 for PH-tau, *n* = 37 for PH-tau-K140A,K141A), which were then multiplied to obtain the nuclear area factor (NAF). The mean NAF across all cells is plotted, with error bars to show SEM. This plot is overlayed with a dot plot indicating NAF values from individual nuclei. Data were analyzed using Welch’s ANOVA, and Games-Howell *post-hoc* test was used for pairwise comparisons The + indicates a significant difference in PH-tau compared to WT-tau (*p* = 0.004) and PH-tau-K140A,K141A (*p* = 0.037).

**FIGURE 5:**
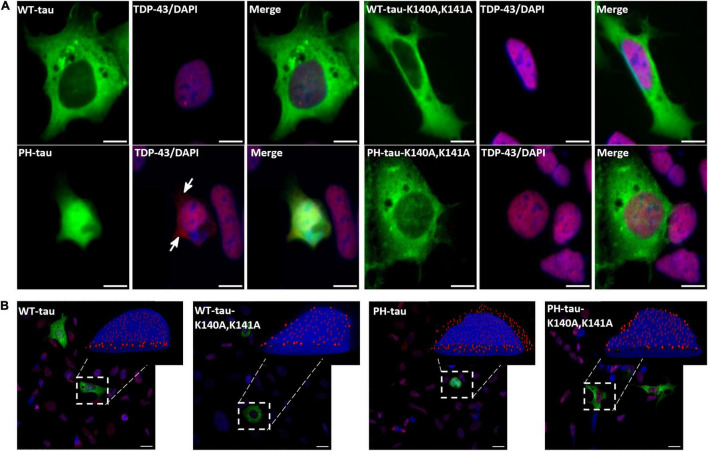
PH-tau-positive cells present mislocalization of TDP-43 to the cytoplasm when PH-tau can enter the nucleus, but not when it is kept in the cytoplasm. **(A)** HEK-293 cells were transfected with WT-tau, PH-tau, WT-tau-K140A,K141A, or PH-tau-K140A,K141A (green) and immunostained for TDP-43 (red). The localization of TDP-43 was determined by looking for co-localization of the TDP-43 stain and DAPI (blue), which presents as purple. The arrows indicate areas of red staining that do not co-localize with DAPI, indicating mislocalization to the cytoplasm. Scale bar indicates 15 μm. **(B)** Overlays of z-stack images taken with a confocal microscope. The inset displays an IMARIS reconstruction of the indicated cell to better display the nuclear or cytoplasmic localization of TDP-43 (red dots). Scale bar indicates 15 μm.

Transfections were performed using either Lipofectamine 2000 (Figures 1–3) or Endofectin Max (Figures 4, 5) according to the manufacturer’s protocol.

### Importin Inhibitor Screening

Transfections were performed as outlined above, but, after 4 h, the media was replaced with fresh media containing either 25 μM ivermectin (Wagstaff et al., 2012; King et al., 2020; Al-Wassiti et al., 2021) or 40 μM importazole (Soderholm et al., 2011; Bird et al., 2013; Kublun et al., 2014; Al-Wassiti et al., 2021; Lee et al., 2021). The cells were incubated for 8 h at 37°C, 5% CO_2_ and then processed for immunocytochemistry.

### Immunocytochemistry

Cells were fixed in 4% paraformaldehyde at room temperature for 10 min 24 h post-transfection. The cells were blocked in a solution of 5% bovine serum albumin in 0.2% PBST for 1 h at room temperature. They were then incubated with primary antibody [anti-α-tubulin (1:500), anti-lamin-B1 (1:100), anti-TDP-43 (1:100), anti-Musashi-1 (1:100), or anti-Ran (1:100)] prepared in blocking buffer overnight at 4°C on a shaker, followed by an anti-mouse or anti-rabbit secondary antibody conjugated to AlexaFluor594 (1:1,000) for 1 h at room temperature. The coverslips were then processed for microscopy.

### Microscopy

Most imaging was done using an Axio Observer Z1 fluorescence microscope (Zeiss) at 40X magnification, digitally zoomed 4 times. All images were collected using the AlexaFluor488 (EGFP), AlexaFluor594 (secondary antibodies), and UV (DAPI) settings. For each condition in every experiment, 10 images were taken randomly from 2 independently transfected and stained sets of cells. For analyzing tau-tubulin interactions, the images were screened for rings and the number of cells with them was counted. For analysis of the nuclear membrane, images were processed in ImageJ to select for the nuclei of tau-positive cells. The mean fluorescence intensity, roundness, and area of the nucleus were recorded. The nuclear area factor was calculated by multiplying the area and roundness of each nucleus. The high magnification (63 × 3) images in Figure 1B were obtained on a Leica SP2 AOBS Confocal Microscope. Z-stacks of these images were imported into IMARIS software for 3D reconstruction.

### Subcellular Fractionation

Cytoplasmic and nuclear fractions were obtained from transfected HEK-293 cells using the NE-PER Nuclear and Cytoplasmic Extraction Kit (Invitrogen #78833) according to the manufacturer’s protocol.

### Western Blotting

Samples were mixed with 4X Laemmli buffer and boiled at 95°C for 5 min. 10% polyacrylamide gels were hand-casted and loaded with 20 μg of sample. The gels were run using a Mini-PROTEAN Tetra Cell (BioRad #1658001) and PowerPac Basic Power Supply (BioRad #1645050) at 100V for approximately 90 min. Gels were prepared for transfer to PVDF membranes, and the transfer was run using a Mini Trans-Blot Module (BioRad #1703935) at 400 mV for 60 min. The membranes were removed and blocked in 5% milk dissolved in TBST for 1 h. They were then incubated overnight with primary antibody [anti-tau (1:500,000), anti-GAPDH (1:1,000), and anti-Histone H3 (1:1,000)] overnight at 4°C on a shaker. The membranes were washed and incubated with secondary antibody [anti-rabbit AlexaFluor600 (1:5,000) and anti-mouse AlexaFluor800 (1:10,000)] for 1 h at room temperature. Imaging was done using a Licor Odyssey 9120 Imaging System. Quantification was done using ImageJ.

### Flow Cytometry

Twenty-four hours post-transfection, cells were detached by trypsinization. The cell suspension was collected into sterile Eppendorf tubes and pelleted by centrifugation at 57 × g for 5 min. Pellets were washed by resuspension in sterile PBS twice, and then resuspended in a final volume of 100 μL of sterile PBS. Immediately prior to analysis, 10 μL of a 10 μg/mL propidium iodide solution was added to each tube, gently mixed, and incubated for 5 min on ice. The samples were analyzed using an Accuri C6 Plus flow cytometer.

### Data Analysis

When quantification was done, data were analyzed using Welch’s ANOVA followed by a Games-Howell *post-hoc* test. All analyses were performed using JASP with α set at *p* = 0.05.

## Results

### PH-Tau Translocates Into the Nucleus *via* an Importin-Mediated Mechanism

Previously, our lab reported that expression of PH-tau was sufficient to mimic the toxicity of hyperphosphorylated tau in cell culture, in Drosophila, and in a transgenic mouse model, including disruption of the microtubule network, cognitive dysfunction, and neurodegeneration (Alonso et al., 2010; Beharry et al., 2013; Di et al., 2016). We have previously observed potential nuclear localization of PH-tau (Alonso et al., 2010; Di et al., 2016). A study looking into the role of nuclear tau found that tau-R406W, which is one of the MAPT mutations associated with familial FTDP-17, was sufficient to cause heterochromatin loss and neurotoxicity in Drosophila (Frost et al., 2014). Therefore, we studied nuclear translocation using both WT-tau and tau-R406W. To do this, HEK-293 cells were transfected with vectors containing EGFP-WT-tau, EGFP-tau-R406W, EGFP-PH-tau, or EGFP-PH-tau-R406W (see Figure 1A, top). We report that PH-tau localizes strongly to the nucleus (Figure 1B, inset; Figures 1C,D, arrow), whereas WT-tau localizes almost entirely to the cytoplasm (Figure 1B, inset; Figures 1C,D). This is seen both with microscopy (Figures 1B,D) and biochemically with subcellular fractionation (Figure 1C). When comparing tau with and without the R406W mutation, we found that tau-R406W did not differ from WT-tau and that PH-tau-R406W did not differ from PH-tau in terms of their nuclear localization, indicating that this translocation is driven by phosphorylation independently of the properties conferred by the R406W mutation (Supplementary Figure 1).

While it has been demonstrated before that tau is able to shuttle from the cytoplasm to the nucleus, the translocation mechanism is unknown. Using NLStradamus prediction software (Nguyen Ba et al., 2009) the presence of an NLS between amino acids 141–178 was proposed (Figure 1A, bottom). This predicted region includes amino acids 140–143 (KKAK) which correspond to the loose consensus monopartite NLS of K(K/R)X(K/R) defined by structural studies (Conti and Kuriyan, 2000; Fontes et al., 2000). In order to investigate whether this sequence serves as an NLS, we performed site-directed mutagenesis to change amino acids 140 and 141 from lysine to alanine (see Figure 1A, top). After transfection with these vectors, we observed that the K140A,K141A mutations did not cause a change in the localization of WT-tau. However, we found that the EGFP signal for PH-tau-K140A,K141A was localized entirely to the cytoplasm, rather than the nucleus (Figure 1B). No distinction can be made between PH-tau-K140A,K141A and PH-tau-R406W-K140A,K141A, suggesting that the R406W mutation does not alter the mechanism of nuclear translocation compared to phosphorylation alone (Supplementary Figure 1). Given the striking nuclear localization observed in PH-tau-expressing cells, this result strongly suggests that amino acids 140–143 of tau serve as an NLS for the nuclear import of PH-tau.

Considering that the consensus monopartite NLS is an importin binding site, we hypothesized that importins were involved in the nuclear translocation of tau. To further investigate this mechanism, HEK-293 were treated with either ivermectin, an inhibitor of importin-α (Wagstaff et al., 2012; Yang et al., 2020), or importazole, an inhibitor of importin-β (Soderholm et al., 2011; Bird et al., 2013). Transfections under drug-free conditions again produced a pattern of strong nuclear localization in PH-tau-expressing cells, but not in the wildtype (Figure 1B and Supplementary Figure 1). When either drug was added to the culture post-transfection, however, PH-tau was found to be largely localized to the cytoplasm (Figures 1B and Supplementary Figure 1). Together, these results indicate that the combined importin-α/importin-β pathway is a likely mechanism for nuclear entry of tau, and suggest that conformational changes induced by phosphorylation in PH-tau are necessary to allow for the import mechanism to function.

### Toxicity of PH-Tau Depends on Its Nuclear Localization

Previously, it was reported that, when PH-tau is expressed in cells, there is a disruption of the microtubule network and cell death (Alonso et al., 2010). To study whether these effects are related to PH-tau nuclear translocation, HEK-293 cells were transfected with our tau and K140A,K141A-tau vectors and immunostained for microtubules. The microtubule bundling effect of tau can be visualized as the presence of a ring-like microtubule structure around the periphery of the cell (Figure 2A, white arrows). This microtubule bundling is a well-known effect of tau expression, and indicates healthy, physiological interaction between tau and the microtubules (Kanai et al., 1989, 1992; Scott et al., 1992). Comparison of tau expressing cells to those not expressing tau demonstrated the presence of these stable ring-like structures when tau was present (Figure 2A, compare white and gold arrows). We observe a significant decrease in the number of cells demonstrating microtubule bundling after transfection with PH-tau compared to WT-tau (Figure 2B). Additionally, PH-tau-K140A,K141A, despite its cytoplasmic localization, failed to induce microtubule bundling (Figure 2B). This suggests that PH-tau cannot bundle microtubules, likely because of a general lack of affinity for tubulin, and not through an indirect mechanism related to its nuclear translocation. There are also no differences between PH-tau and PH-tau-R406W (Supplementary Figure 2). PH-tau-K140A,K141A, and PH-tau-R406W-K140A,K141A both localize to the cytoplasm, and both fail to induce microtubule bundling (Figure 2B and Supplementary Figure 2), corroborating our previous report that phosphorylation at these sites, and not the R406W mutation, interrupts tau-microtubule interactions (Alonso et al., 2010).

To better understand whether nuclear PH-tau and cytosolic PH-tau differ in their toxicity and contribution to cell death we transfected HEK-293 cells with different tau vectors as above, and then processed them for flow cytometry with propidium iodide (PI). Graphs indicating the gating mechanism can be found in Supplementary Figure 3. Increased PI fluorescence is indicative of dead and dying cells. The results demonstrate that PH-Tau, but not WT-tau, expression results in increased cell death as indicated by increased proportion of PI-positive cells (Figures 3A,B; left; for tau-R406W, see Supplementary Figure 4). Further, prevention of PH-tau entry into the nucleus by mutating the NLS site reduces the percentage of dead and dying cells, strongly suggesting that the nuclear localization of PH-tau is necessary for its cellular toxicity (Figures 3A,B; left; for tau-R406W, see Supplementary Figure 4). Since our transfected cells also express EGFP, we were able to quantify the number of tau-positive cells within each population, as well. This analysis reveals a significant decrease in the percentage of EGFP-positive cells in the PH-tau-transfected populations compared to those of WT-tau and PH-tau-K140A,K141A (Figures 3A,B; right; for tau-R406W, see Supplementary Figure 4). This result is in line with that of the PI analysis, synergistically showing that the number of viable tau-expressing cells is reduced after transfection with PH-tau, but not WT-tau or PH-tau-K140A,K141A.

### Nuclear Translocation of PH-Tau Results in Changes to the Nuclear Lamina and Mislocalization of TDP-43

Disruptions of the nuclear envelope have previously been associated both with tauopathy (Frost et al., 2016; Montalbano et al., 2019) and with dysregulation of nucleocytoplasmic transport (de Vos et al., 2011; Ferri et al., 2017). As such, we sought to determine whether the observed toxicity of nuclear PH-tau was accompanied by changes to the nuclear envelope. The nuclear envelope was visualized by immunostaining tau-transfected cells for lamin-B1 (Figure 4A). We observe a general change in the nuclear morphology of PH-tau-expressing cells, with the majority having irregular shapes and visible invagination of the nuclear lamina (Figure 4A). We report a significant decrease in the mean fluorescence intensity of lamin only in PH-tau-positive cells, suggesting a decrease in lamin-B1 as a result of PH-tau expression in the nucleus (Figure 4B, left; for tau-R406W, see Supplementary Figure 5). Altered nuclear morphology is quantitatively characterized by the computation of the nuclear area factor, which reveals that PH-tau-positive cells have significantly reduced area and roundness compared to both WT-tau-positive cells and cells expressing K140A,K141A-tau (Figure 4B, right; for tau-R406W, see Supplementary Figure 5).

The involvement of TDP-43 in multiple tauopathies is well-established, and recent work has suggested an interaction between tau and TDP-43 (Montalbano et al., 2020a). Additionally, tau aggregates have been shown to mislocalize several proteins, including the RNA-binding protein Musashi (Montalbano et al., 2020b) and Ran (Eftekharzadeh et al., 2018). Given this, we used immunocytochemistry to probe the localization of these proteins. The expression of WT-tau does not alter the expected subcellular localization of TDP-43 (Figures 5A,B). However, the expression of PH-tau changes the localization of TDP-43 to the cytoplasm (Figure 5A, arrows; Figure 5B, insets; for tau-R406W, see Supplementary Figure 6). Preventing nuclear import of tau restores the strong pattern of nuclear localization (Figures 5A,B). Interestingly, immunostains of Ran (Supplementary Figure 8) and Musashi-1 (Supplementary Figure 7) show no significant differences in localization. These results suggest that there is a relationship between the subcellular localization of tau and of TDP-43. Nuclear localization of PH-tau may interfere with the correct sorting of TDP-43 which could lead to toxic phenotypes observed in cells expressing the pathological tau.

## Discussion

Much research has been done over the last decade to begin identifying the physiological functions of nuclear tau. Nuclear tau has been shown to be predominantly non-phosphorylated (Greenwood and Johnson, 1995), as phosphorylation could destabilize its interactions with DNA or with mRNA-processing machinery (Lu et al., 2013; Camero et al., 2014; Qi et al., 2015; Baquero et al., 2019). In previous work, we observed nuclear localization of PH-tau (Alonso et al., 2010) which led us to consider the role of tau phosphorylation state in nuclear translocation. PH-tau is well-suited to studying tau hyperphosphorylation because it mimics the effects of AD P-tau (Alonso et al., 2010; Beharry et al., 2013; Di et al., 2016). Additionally, because PH-tau is pseudophosphorylated, the conformational changes induced by mutating Ser/Thr residues to Glu are not reversible. Using PH-tau to study the mechanism of tau entry into the nucleus and its subsequent toxicity is important to determine mechanisms of neurodegeneration in tauopathies.

In this work, we transiently transfected HEK-293 cells with different EGFP-fused forms of tau. Once again, we observed robust nuclear localization of PH-tau (Figures 1B–D) and established a direct link between tau phosphorylation and nuclear translocation. The importance of phosphorylation has been long suggested, as phospho-tau is found in the nucleus, but the phosphorylation occurs in the cytoplasm (Greenwood and Johnson, 1995). While the ability of tau to shuttle into the nucleus has been previously demonstrated, the importance of tau phosphorylation for its nuclear translocation was not directly established (Sultan et al., 2011). Given the intrinsically disordered nature of tau, it is highly possible that the addition of negative charges at phosphorylation sites can trigger conformational changes. Thus, we propose that phosphorylation at the chosen sites can lead to conformational changes in the folding of tau which can expose the NLS to importins. Importantly, we also transfected cells with tau containing the R406W mutation (Supplementary Figure 1). This mutation is sufficient to trigger neurodegeneration in FTDP-17 patients, and previous work has suggested that this is the result of conformational changes in tau due to the R406W mutation (Jicha et al., 1999). Additionally, tau-R406W was shown to be sufficient to cause heterochromatin loss and neurotoxicity in a Drosophila model (Frost et al., 2014). Our results indicate that any conformational change induced by R406W is not sufficient to cause nuclear translocation, but that phosphorylation of both WT-tau and tau-R406W allows for nuclear translocation.

In order to investigate the mechanism of nuclear import, we looked for possible NLS sites within the tau sequence. Using the freely available NLS prediction software NLStradamus (Nguyen Ba et al., 2009), we identified amino acids 141–178 in the primary sequence of tau as a possible NLS (Figure 1A, bottom). Our attention was drawn to amino acids 140–143 (KKAK), which correspond to a consensus monopartite NLS. Interestingly, other modeling software, including cNLS mapper, SeqNLS, NLSPredict, and NucPred, failed to predict this NLS. However, disruption of this putative NLS strongly changes PH-tau localization to the cytoplasm, indicating its importance for nuclear import of tau (Figures 1B,C). It is critical to note that this sequence of amino acids remains intact in WT-tau. Therefore, amino acids 140–143 are necessary, but not sufficient, for nuclear translocation of tau. This reinforces our suggestion that phosphorylation of tau triggers conformational changes that expose the NLS, allowing for translocation into the nucleus. It also fits the long-standing model where tau phosphorylation occurs in the cytoplasm, with subsequent dephosphorylation occurring in the nucleus, presumably after translocation (Greenwood and Johnson, 1995). We further explore the mechanism of tau nuclear import by using pharmacological inhibitors of importins. The data show that inhibiting either importin-α or importin-β was sufficient to block the translocation of PH-tau into the nucleus (Figure 1D). This is consistent with the importin-α/importin-β mechanism as consensus monopartite NLSs are accepted to be importin-α binding sites, which is then followed by the binding of importin-β to mediate nuclear import.

**FIGURE 6:**
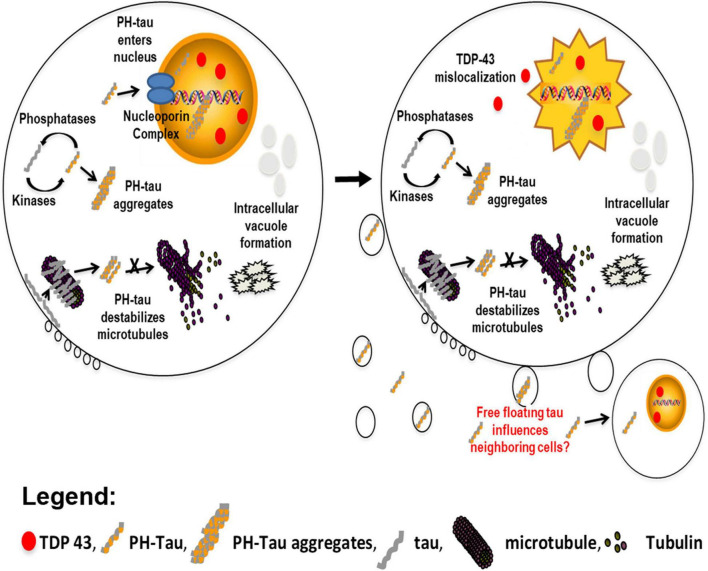
Model proposing the independent actions of pathological tau within a cell. In the cytoplasm, an imbalance of kinase and phosphatase activity contributes to the hyperphosphorylation of tau. This hyperphosphorylated tau can sequester normal tau to form aggregates. This behavior results in destabilization of the microtubule network and the formation of intracellular vacuoles (left). We have now demonstrated that PH-tau is able to translocate into the nucleus (left & right). This results in disruption of the nuclear lamina and mislocalization of the nuclear protein TDP-43 (red ovals) to the cytoplasm, ultimately inducing cellular death (right). It is currently hypothesized that PH-tau can propagate in a prion-like manner. We have shown that monomeric tau can be uptaken by healthy neurons (Morozova et al., 2019), and other reports have seen the same for oligomeric tau (Guo and Lee, 2013; Calafate et al., 2015; Evans et al., 2018). This uptake of PH-tau by healthy cells could spread the cytoskeletal and nuclear pathologies.

We have previously reported that PH-tau could disrupt the microtubule network (Alonso et al., 2010). However, with the ability to block nuclear translocation, we can now investigate whether there is an additional indirect effect of tau on microtubules due to its nuclear translocation. We found that, regardless of whether PH-tau could enter the nucleus, there was significantly reduced tau-tubulin interaction (Figure 2B). While PH-tau-K140A,K141A was unable to enter the nucleus, the loss of microtubule bundling was not recovered, indicating that the two phenotypes are not related. Given these results, we propose that hyperphosphorylated tau has multiple gains of toxic function within cells (Figure 6). One such function is the disruption of the microtubule network and axonal transport, as described previously (LaPointe et al., 2009; Kanaan et al., 2011). Another involves tau translocation into the nucleus, which we associate here with the disruption of NCT and increased cell death. This is backed by the observation that reduced microtubule bundling is the only effect of PH-tau expression that is not spared by blocking nuclear translocation of PH-tau. Overall, we suggest that microtubule disruption and nuclear dysfunction, both triggered by tau phosphorylation and subsequent conformational changes, are distinct mechanisms of cellular toxicity in tauopathies.

In a similar manner, we have previously reported that the expression of PH-tau is toxic to cells (Alonso et al., 2010), but are now in the position to distinguish whether cytosolic PH-tau and nuclear PH-tau differ in their toxicity. We did this using flow cytometry with PI. We report a significant increase in the number of dead and dying cells in the PH-tau-transfected population compared to the WT-tau-transfected population (Figures 3A,B; left; for tau-R406W, see Supplementary Figure 4). This increase is not seen, however, in cells transfected with PH-tau-K140A,K141A (Figures 3A,B; left). Measuring EGFP signal by flow cytometry reveals a concomitant decrease in the number of EGFP-positive cells in the PH-tau group compared to either WT or PH-tau-K140A,K141A (Figures 3A,B; right). An attractive mechanism for investigating this toxicity of nuclear tau is the disruption of nuclear membrane integrity and nucleocytoplasmic transport, which have been implicated in a number of neurodegenerative diseases, including frontotemporal dementia, Huntington’s disease, AD, and ALS. Indeed, we found that cells expressing PH-tau had highly irregular nuclei, with abnormal shapes (Figure 4B, right) and invaginations of the nuclear lamina (Figure 4A, arrow). This coincides with reports of irregular nuclei in postmortem sections from patients with neurodegenerative diseases (Gil et al., 2020). The reduction in lamin fluorescence intensity (Figure 4B, left) in these cells points toward a reduction in the lamin content of the nuclear envelope, but that cannot be definitively determined from the experiments reported here.

We also report mislocalization of TDP-43 to the cytoplasm in PH-tau-expressing cells, but not in WT-tau-expressing or PH-tau-K140A,K141A-expressing cells (Figure 5A, arrows; Figure 5B, insets). As nuclear tau has been seen in the brains of tauopathy patients (Metuzals et al., 1988), it is possible that mislocalization of TDP-43 into the cytoplasm could be a downstream consequence of pathological tau nuclear localization. It is known that TDP-43 translocates into the nucleus through the importin-α/importin-β pathway (Nishimura et al., 2010), so our results here may indicate a general interruption of NCT where a high level of PH-tau expression interrupts the equilibrium of importin binding to other substrates. However, further study is needed to characterize the mechanistic relationship between nuclear PH-tau and TDP-43. In particular, a major question raised by this result is how nuclear tau could trigger mislocalization of TDP-43. A striking feature of both tau (Eftekharzadeh et al., 2018) and TDP-43 (Chou et al., 2018) pathologies is their ability to form aggregates that can sequester nucleoporins and disrupt NCT. The previously reported disruption of NCT by cytosolic tau aggregates (Eftekharzadeh et al., 2018) was accompanied by mislocalization of Ran. However, we do not observe mislocalization of Ran (Supplementary Figure 8), only of TDP-43 (Figures 5A,B). It is possible then, that cytosolic and nuclear tau interfere with NCT in different ways. Either way, the core mechanism underlying this disruption of NCT remains unclear. Several studies have suggested that aberrant phase transition of proteins with intrinsically disordered regions may serve as a starting point for the toxicity of aggregation-prone proteins (Molliex et al., 2015; Murakami et al., 2015; Patel et al., 2015). Notably, nucleoporins (Denning et al., 2003; Frey et al., 2006), TDP-43 (Babinchak et al., 2019), and tau (Wegmann et al., 2018) all contain intrinsically disordered regions that undergo such phase transitions. It will be critical to develop a better understanding of these liquid-phase interactions, how post-translational modification of tau can modulate them, and how they contribute to the disruption of NCT in tauopathies.

In conclusion, our data demonstrate that tau pseudophosphorylation at residues S199, T212, T231, and S262 allows tau to enter the nucleus through the classic importin-α/importin-β pathway and that this nuclear localization produces toxic phenotypes reminiscent of tauopathies. PH-tau, but not WT-tau, translocates into the nucleus (Figure 6, left). We identify a nuclear localization sequence within tau and demonstrate that it is necessary, but not sufficient, for nuclear translocation of tau. Thus, phosphorylation at appropriate sites seems likely to cause conformational changes that enable the NLS to function. When PH-tau enters the nucleus, we observe a high degree of cell death, as well as disruption of the nuclear lamina and mislocalization of TDP-43 to the cytoplasm (Figure 6, right). All of these changes are prevented by mutating the NLS and preventing the nuclear translocation of PH-tau. PH-tau fails to bind microtubules, but this change also occurs with PH-tau-K140A,K141A. These results have implications both for further research into the biochemistry of nuclear tau and for our understanding of the pathogenesis of many neurodegenerative diseases. We identify a mechanism for the nuclear import of tau that depends on its phosphorylation, opening the door for further investigation into the kinetics and regulation of this shuttling. Additionally, we demonstrate that the presence of phosphorylated tau in the nucleus produces tauopathy-like phenotypes, which implicates nuclear tau in neurodegenerative disease. We propose that nuclear hyperphosphorylated tau acts alongside the classic microtubule-mediated mechanism to harm the cell, but further research will be needed to understand the exact mechanisms underlying the toxicity of nuclear tau, and how its actions may differ from cytosolic tau.

## Data Availability Statement

The original contributions presented in the study are included in the article/Supplementary Material, further inquiries can be directed to the corresponding author/s.

## Author Contributions

CC, AA, and LC generated plasmids and performed site-directed mutagenesis. VM and RC performed the experiments. RC, AA, and LC designed the experiments, contributed to critical discussion, and wrote the manuscript. All authors contributed to the article and approved the submitted version.

## Conflict of Interest

The authors declare that the research was conducted in the absence of any commercial or financial relationships that could be construed as a potential conflict of interest.

## Publisher’s Note

All claims expressed in this article are solely those of the authors and do not necessarily represent those of their affiliated organizations, or those of the publisher, the editors and the reviewers. Any product that may be evaluated in this article, or claim that may be made by its manufacturer, is not guaranteed or endorsed by the publisher.

## References

[B1] Alonso A. C. Grundke-Iqbal I. Iqbal K. (1996). Alzheimer’s disease hyperphosphorylated tau sequesters normal tau into tangles of filaments and disassembles microtubules. *Nat. Med.* 2 783–787. 10.1038/nm0796-783 8673924

[B2] Alonso A. C. Zaidi T. Grundke-Iqbal I. Iqbal K. (1994). Role of abnormally phosphorylated tau in the breakdown of microtubules in Alzheimer disease. *Proc. Natl. Acad. Sci. U S A.* 91 5562–5566. 10.1073/pnas.91.12.5562 8202528 PMC44036

[B3] Alonso A. D. Di Clerico J. Li B. Corbo C. P. Alaniz M. E. Grundke-Iqbal I. (2010). Phosphorylation of tau at Thr212, Thr231, and Ser262 combined causes neurodegeneration. *J. Biol. Chem.* 285 30851–30860. 10.1074/jbc.M110.110957 20663882 PMC2945578

[B4] Alonso A. D. Grundke-Iqbal I. Barra H. S. Iqbal K. (1997). Abnormal phosphorylation of tau and the mechanism of Alzheimer neurofibrillary degeneration: sequestration of microtubule-associated proteins 1 and 2 and the disassembly of microtubules by the abnormal tau. *Proc. Natl. Acad. Sci. U S A.* 94 298–303. 10.1073/pnas.94.1.298 8990203 PMC19321

[B5] Alonso A. D. C. Li B. Grundke-Iqbal I. Iqbal K. (2006). Polymerization of hyperphosphorylated tau into filaments eliminates its inhibitory activity. *Proc. Natl. Acad. Sci.* 103 8864–8869. 10.1073/pnas.0603214103 16735465 PMC1482669

[B6] Al-Wassiti H. A. Thomas D. R. Wagstaff K. M. Fabb S. A. Jans D. A. Johnston A. P. (2021). Adenovirus Terminal Protein Contains a Bipartite Nuclear Localisation Signal Essential for Its Import into the Nucleus. *Int. J. Mol. Sci.* 22:3310. 10.3390/ijms22073310 33804953 PMC8036708

[B7] Arriagada P. V. Growdon J. H. Hedley-Whyte E. T. Hyman B. T. (1992). Neurofibrillary tangles but not senile plaques parallel duration and severity of Alzheimer’s disease. *Neurology* 42 631–639. 10.1212/WNL.42.3.631 1549228

[B8] Babinchak W. M. Haider R. Dumm B. K. Sarkar P. Surewicz K. Choi J.-K. (2019). The role of liquid-liquid phase separation in aggregation of the TDP-43 low-complexity domain. *J. Biol. Chem.* 294 6306–6317. 10.1074/jbc.RA118.007222 30814253 PMC6484124

[B9] Baquero J. Varriano S. Ordonez M. Kuczaj P. Murphy M. R. Aruggoda G. (2019). Nuclear Tau, p53 and Pin1 Regulate PARN-Mediated Deadenylation and Gene Expression. *Front. Mol. Neurosci.* 12:242. 10.3389/fnmol.2019.00242 31749682 PMC6843027

[B10] Beharry C. Alaniz M. E. Alonso Adel C. (2013). Expression of Alzheimer-Like Pathological Human Tau Induces a Behavioral Motor and Olfactory Learning Deficit in Drosophila melanogaster. *J. Alzheimer’s Dis.* 37 539–550. 10.3233/JAD-130617 23948901

[B11] Bird S. L. Heald R. Weis K. (2013). RanGTP and CLASP1 cooperate to position the mitotic spindle. *Mol. Biol. Cell* 24 2506–2514. 10.1091/mbc.e13-03-0150 23783028 PMC3744954

[B12] Calafate S. Buist A. Miskiewicz K. Vijayan V. Daneels G. de Strooper B. (2015). Synaptic Contacts Enhance Cell-to-Cell Tau Pathology Propagation. *Cell Rep.* 11 1176–1183. 10.1016/j.celrep.2015.04.043 25981034

[B13] Camero S. Benítez M. J. Cuadros R. Hernández F. Ávila J. Jiménez J. S. (2014). Thermodynamics of the Interaction between Alzheimer’s Disease Related Tau Protein and DNA. *PLoS One* 9:e104690. 10.1371/journal.pone.0104690 25126942 PMC4134230

[B14] Carmody S. R. Wente S. R. (2009). mRNA nuclear export at a glance. *J. Cell Sci.* 122 1933–1937. 10.1242/jcs.041236 19494120 PMC2723150

[B15] Cevher M. A. Zhang X. Fernandez S. Kim S. Baquero J. Nilsson P. (2010). Nuclear deadenylation/polyadenylation factors regulate 3’ processing in response to DNA damage. *EMBO J.* 29 1674–1687. 10.1038/emboj.2010.59 20379136 PMC2876964

[B16] Chou C.-C. Zhang Y. Umoh M. E. Vaughan S. W. Lorenzini I. Liu F. (2018). TDP-43 pathology disrupts nuclear pore complexes and nucleocytoplasmic transport in ALS/FTD. *Nat. Neurosci.* 21 228–239. 10.1038/s41593-017-0047-3 29311743 PMC5800968

[B17] Conti E. Kuriyan J. (2000). Crystallographic analysis of the specific yet versatile recognition of distinct nuclear localization signals by karyopherin alpha. *Structure* 8 329–338. 10.1016/s0969-2126(00)00107-6 10745017

[B18] Corces V. G. Manso R. De La Torre J. Avila J. Nasr A. Wiche G. (1980). Effects of DNA on microtubule assembly. *Eur. J. Biochem.* 105 7–16. 10.1111/j.1432-1033.1980.tb04468.x 6154575

[B19] Corces V. G. Salas J. Salas M. L. Avila J. (1978). Binding of microtubule proteins to DNA: specificity of the interaction. *Eur. J. Biochem.* 86 473–479. 10.1111/j.1432-1033.1978.tb12330.x 207527

[B20] de Vos W. H. Houben F. Kamps M. Malhas A. Verheyen F. Cox J. (2011). Repetitive disruptions of the nuclear envelope invoke temporary loss of cellular compartmentalization in laminopathies. *Hum. Mol. Genet.* 20 4175–4186. 10.1093/hmg/ddr344 21831885

[B21] Denning D. P. Patel S. S. Uversky V. Fink A. L. Rexach M. (2003). Disorder in the nuclear pore complex: the FG repeat regions of nucleoporins are natively unfolded. *Proc. Natl. Acad. Sci. U S A.* 100 2450–2455. 10.1073/pnas.0437902100 12604785 PMC151361

[B22] Devany E. Zhang X. Park J. Y. Tian B. Kleiman F. E. (2013). Positive and negative feedback loops in the p53 and mRNA 3’ processing pathways. *Proc. Natl. Acad. Sci.* 110 3351–3356. 10.1073/pnas.1212533110 23401530 PMC3587245

[B23] Di J. Cohen L. S. Corbo C. P. Phillips G. R. El Idrissi A. Alonso A. D. (2016). Abnormal tau induces cognitive impairment through two different mechanisms: synaptic dysfunction and neuronal loss. *Sci. Rep.* 6:20833. 10.1038/srep20833 26888634 PMC4757872

[B24] Eftekharzadeh B. Daigle J. G. Kapinos L. E. Coyne A. Schiantarelli J. Carlomagno Y. (2018). Tau Protein Disrupts Nucleocytoplasmic Transport in Alzheimer’s Disease. *Neuron* 99 925–940.e7. 10.1016/j.neuron.2018.07.039 30189209 PMC6240334

[B25] Evans L. D. Wassmer T. Fraser G. Smith J. Perkinton M. Billinton A. (2018). Extracellular Monomeric and Aggregated Tau Efficiently Enter Human Neurons through Overlapping but Distinct Pathways. *Cell Rep.* 22 3612–3624. 10.1016/j.celrep.2018.03.021 29590627 PMC5896171

[B26] Ferri G. Storti B. Bizzarri R. (2017). Nucleocytoplasmic transport in cells with progerin-induced defective nuclear lamina. *Biophys. Chem.* 229 77–83. 10.1016/j.bpc.2017.06.003 28712764

[B27] Fontes M. R. M. Teh T. Kobe B. (2000). Structural basis of recognition of monopartite and bipartite nuclear localization sequences by mammalian importin-α11Edited by K. Nagai. *J. Mol. Biol.* 297 1183–1194. 10.1006/jmbi.2000.3642 10764582

[B28] Freibaum B. D. Lu Y. Lopez-Gonzalez R. Kim N. C. Almeida S. Lee K.-H. (2015). GGGGCC repeat expansion in C9orf72 compromises nucleocytoplasmic transport. *Nature* 525 129–133. 10.1038/nature14974 26308899 PMC4631399

[B29] Frey S. Richter R. P. Görlich D. (2006). FG-Rich Repeats of Nuclear Pore Proteins Form a Three-Dimensional Meshwork with Hydrogel-Like Properties. *Science* 314 815–817. 10.1126/science.1132516 17082456

[B30] Frost B. Bardai F. H. Feany M. B. (2016). Lamin Dysfunction Mediates Neurodegeneration in Tauopathies. *Curr. Biol.* 26 129–136. 10.1016/j.cub.2015.11.039 26725200 PMC4713335

[B31] Frost B. Hemberg M. Lewis J. Feany M. B. (2014). Tau promotes neurodegeneration through global chromatin relaxation. *Nat. Neurosci.* 17 357–366. 10.1038/nn.3639 24464041 PMC4012297

[B32] Gil L. Niño S. A. Chi-Ahumada E. Rodríguez-Leyva I. Guerrero C. Rebolledo A. B. (2020). Perinuclear Lamin A and Nucleoplasmic Lamin B2 Characterize Two Types of Hippocampal Neurons through Alzheimer’s Disease Progression. *Int. J. Mol. Sci.* 21:1841. 10.3390/ijms21051841 32155994 PMC7084765

[B33] Greenwood J. A. Johnson G. V. W. (1995). Localization and in Situ Phosphorylation State of Nuclear Tau. *Exp. Cell Res.* 220 332–337. 10.1006/excr.1995.1323 7556441

[B34] Guo J. L. Lee V. M. Y. (2013). Neurofibrillary tangle-like tau pathology induced by synthetic tau fibrils in primary neurons over-expressing mutant tau. *FEBS Lett.* 587 717–723. 10.1016/j.febslet.2013.01.051 23395797 PMC3678381

[B35] Hutton M. Lendon C. L. Rizzu P. Baker M. Froelich S. Houlden H. (1998). Association of missense and 5’-splice-site mutations in tau with the inherited dementia FTDP-17. *Nature* 393 702–705. 10.1038/31508 9641683

[B36] Jicha G. A. Rockwood J. M. Berenfeld B. Hutton M. Davies P. (1999). Altered conformation of recombinant frontotemporal dementia-17 mutant tau proteins. *Neurosci. Lett.* 260 153–156. 10.1016/s0304-3940(98)00980-x 10076890

[B37] Jovičić A. Mertens J. Boeynaems S. Bogaert E. Chai N. Yamada S. B. (2015). Modifiers of C9orf72 dipeptide repeat toxicity connect nucleocytoplasmic transport defects to FTD/ALS. *Nat. Neurosci.* 18 1226–1229. 10.1038/nn.4085 26308983 PMC4552077

[B38] Kanaan N. M. Morfini G. A. LaPointe N. E. Pigino G. F. Patterson K. R. Song Y. (2011). Pathogenic forms of tau inhibit kinesin-dependent axonal transport through a mechanism involving activation of axonal phosphotransferases. *J. Neurosci.* 31 9858–9868. 10.1523/JNEUROSCI.0560-11.2011 21734277 PMC3391724

[B39] Kanai Y. Chen J. Hirokawa N. (1992). Microtubule bundling by tau proteins in vivo: analysis of functional domains. *EMBO J.* 11 3953–3961. 10.1002/j.1460-2075.1992.tb05489.x 1396588 PMC556906

[B40] Kanai Y. Takemura R. Oshima T. Mori H. Ihara Y. Yanagisawa M. (1989). Expression of multiple tau isoforms and microtubule bundle formation in fibroblasts transfected with a single tau cDNA. *J. Cell Biol.* 109 1173–1184. 10.1083/jcb.109.3.1173 2504728 PMC2115758

[B41] King C. R. Tessier T. M. Dodge M. J. Weinberg J. B. Mymryk J. S. (2020). Inhibition of Human Adenovirus Replication by the Importin α/β1 Nuclear Import Inhibitor Ivermectin. *J. Virol.* 94 e00710–20. 10.1128/JVI.00710-20 32641484 PMC7459547

[B42] Klein H.-U. McCabe C. Gjoneska E. Sullivan S. E. Kaskow B. J. Tang A. (2019). Epigenome-wide study uncovers large-scale changes in histone acetylation driven by tau pathology in aging and Alzheimer’s human brains. *Nat. Neurosci.* 22 37–46. 10.1038/s41593-018-0291-1 30559478 PMC6516529

[B43] Kublun I. Ehm P. Brehm M. A. Nalaskowski M. M. (2014). Efficacious inhibition of Importin α/β-mediated nuclear import of human inositol phosphate multikinase. *Biochimie* 102 117–123. 10.1016/j.biochi.2014.03.001 24632208

[B44] LaPointe N. E. Morfini G. Pigino G. Gaisina I. N. Kozikowski A. P. Binder L. I. (2009). The amino terminus of tau inhibits kinesin-dependent axonal transport: implications for filament toxicity. *J. Neurosci. Res.* 87 440–451. 10.1002/jnr.21850 18798283 PMC2739042

[B45] Lee J. Le L. T. H. L. Kim E. Lee M. J. (2021). Formation of Non-Nucleoplasmic Proteasome Foci during the Late Stage of Hyperosmotic Stress. *Cells* 10:2493. 10.3390/cells10092493 34572142 PMC8467775

[B46] Lindwall G. Cole R. D. (1984). Phosphorylation affects the ability of tau protein to promote microtubule assembly. *J. Biol. Chem.* 259 5301–5305. 6425287

[B47] Lu Y. He H.-J. Zhou J. Miao J.-Y. Lu J. He Y.-G. (2013). Hyperphosphorylation results in tau dysfunction in DNA folding and protection. *J. Alzheimers Dis.* 37 551–563. 10.3233/JAD-130602 24064506

[B48] Malpetti M. Passamonti L. Rittman T. Jones P. S. Vázquez Rodríguez P. Bevan-Jones W. R. (2020). Neuroinflammation and Tau Colocalize in vivo in Progressive Supranuclear Palsy. *Ann. Neurol.* 88 1194–1204. 10.1002/ana.25911 32951237 PMC7116392

[B49] Mandelkow E. M. Biernat J. Drewes G. Gustke N. Trinczek B. Mandelkow E. (1995). Tau domains, phosphorylation, and interactions with microtubules. *Neurobiol. Aging* 16 355–62; discussion362–3. 10.1016/0197-4580(95)00025-a 7566345

[B50] Mansuroglu Z. Benhelli-Mokrani H. Marcato V. Sultan A. Violet M. Chauderlier A. (2016). Loss of Tau protein affects the structure, transcription and repair of neuronal pericentromeric heterochromatin. *Sci. Rep.* 6:33047. 10.1038/srep33047 27605042 PMC5015075

[B51] Metuzals J. Robitaille Y. Houghton S. Gauthier S. Leblanc R. (1988). Paired helical filaments and the cytoplasmic-nuclear interface in Alzheimer’s disease. *J. Neurocytol.* 17 827–833. 10.1007/BF01216709 3230400

[B52] Molliex A. Temirov J. Lee J. Coughlin M. Kanagaraj A. P. Kim H. J. (2015). Phase separation by low complexity domains promotes stress granule assembly and drives pathological fibrillization. *Cell* 163 123–133. 10.1016/j.cell.2015.09.015 26406374 PMC5149108

[B53] Montalbano M. McAllen S. Cascio F. l Sengupta U. Garcia S. Bhatt N. (2020a). TDP-43 and Tau Oligomers in Alzheimer’s Disease, Amyotrophic Lateral Sclerosis, and Frontotemporal Dementia. *Neurobiol. Dis.* 146:105130. 10.1016/j.nbd.2020.105130 33065281 PMC7703712

[B54] Montalbano M. McAllen S. Puangmalai N. Sengupta U. Bhatt N. Johnson O. D. (2020b). RNA-binding proteins Musashi and tau soluble aggregates initiate nuclear dysfunction. *Nat. Commun.* 11:4305. 10.1038/s41467-020-18022-6 32855391 PMC7453003

[B55] Montalbano M. McAllen S. Sengupta U. Puangmalai N. Bhatt N. Ellsworth A. (2019). Tau oligomers mediate aggregation of RNA-binding proteins Musashi1 and Musashi2 inducing Lamin alteration. *Aging Cell* 18:e13035. 10.1111/acel.13035 31532069 PMC6826126

[B56] Morozova V. Cohen L. S. Makki A. E.-H. Shur A. Pilar G. el Idrissi A. (2019). Normal and Pathological Tau Uptake Mediated by M1/M3 Muscarinic Receptors Promotes Opposite Neuronal Changes. *Front. Cell. Neurosci.* 13:403. 10.3389/fncel.2019.00403 31555098 PMC6737038

[B57] Murakami T. Qamar S. Lin J. Q. Schierle G. S. K. Rees E. Miyashita A. (2015). ALS/FTD Mutation-Induced Phase Transition of FUS Liquid Droplets and Reversible Hydrogels into Irreversible Hydrogels Impairs RNP Granule Function. *Neuron* 88 678–690. 10.1016/j.neuron.2015.10.030 26526393 PMC4660210

[B58] Nelson P. T. Alafuzoff I. Bigio E. H. Bouras C. Braak H. Cairns N. J. (2012). Correlation of Alzheimer Disease Neuropathologic Changes With Cognitive Status: A Review of the Literature. *J. Neuropathol. Exp. Neurol.* 71 362–381. 10.1097/NEN.0b013e31825018f7 22487856 PMC3560290

[B59] Nguyen Ba A. N. Pogoutse A. Provart N. Moses A. M. (2009). NLStradamus: a simple Hidden Markov Model for nuclear localization signal prediction. *BMC Bioinform.* 10:202. 10.1186/1471-2105-10-202 19563654 PMC2711084

[B60] Nishimura A. L. Župunski V. Troakes C. Kathe C. Fratta P. Howell M. (2010). Nuclear import impairment causes cytoplasmic trans-activation response DNA-binding protein accumulation and is associated with frontotemporal lobar degeneration. *Brain* 133 1763–1771. 10.1093/brain/awq111 20472655

[B61] Patel A. Lee H. O. Jawerth L. Maharana S. Jahnel M. Hein M. Y. (2015). A Liquid-to-Solid Phase Transition of the ALS Protein FUS Accelerated by Disease Mutation. *Cell* 162 1066–1077. 10.1016/j.cell.2015.07.047 26317470

[B62] Poorkaj P. Bird T. D. Wijsman E. Nemens E. Garruto R. M. Anderson L. (1998). Tau is a candidate gene for chromosome 17 frontotemporal dementia. *Ann. Neurol.* 43 815–825. 10.1002/ana.410430617 9629852

[B63] Qi H. Cantrelle F.-X. Benhelli-Mokrani H. Smet-Nocca C. Buée L. Lippens G. (2015). Nuclear Magnetic Resonance Spectroscopy Characterization of Interaction of Tau with DNA and Its Regulation by Phosphorylation. *Biochemistry* 54 1525–1533. 10.1021/bi5014613 25623359

[B64] Scott C. W. Klika A. B. Lo M. M. Norris T. E. Caputo C. B. (1992). Tau protein induces bundling of microtubules in vitro: comparison of different tau isoforms and a tau protein fragment. *J. Neurosci. Res.* 33 19–29. 10.1002/jnr.490330104 1360542

[B65] Soderholm J. F. Bird S. L. Kalab P. Sampathkumar Y. Hasegawa K. Uehara-Bingen M. (2011). Importazole, a small molecule inhibitor of the transport receptor importin-β. *ACS Chem. Biol.* 6 700–708. 10.1021/cb2000296 21469738 PMC3137676

[B66] Sultan A. Nesslany F. Violet M. Bégard S. Loyens A. Talahari S. (2011). Nuclear tau, a key player in neuronal DNA protection. *J. Biol. Chem.* 286 4566–4575. 10.1074/jbc.M110.199976 21131359 PMC3039398

[B67] Wagstaff K. M. Sivakumaran H. Heaton S. M. Harrich D. Jans D. A. (2012). Ivermectin is a specific inhibitor of importin α/β-mediated nuclear import able to inhibit replication of HIV-1 and dengue virus. *Biochem. J.* 443 851–856. 10.1042/BJ20120150 22417684 PMC3327999

[B68] Wegmann S. Eftekharzadeh B. Tepper K. Zoltowska K. M. Bennett R. E. Dujardin S. (2018). Tau protein liquid-liquid phase separation can initiate tau aggregation. *EMBO J.* 37:e98049. 10.15252/embj.201798049 29472250 PMC5881631

[B69] Weingarten M. D. Lockwood A. H. Hwo S. Y. Kirschner M. W. (1975). A protein factor essential for microtubule assembly. *Proc. Natl. Acad. Sci.* 72 1858–1862. 10.1073/pnas.72.5.1858 1057175 PMC432646

[B70] Yang S. N. Y. Atkinson S. C. Wang C. Lee A. Bogoyevitch M. A. Borg N. A. (2020). The broad spectrum antiviral ivermectin targets the host nuclear transport importin α/β1 heterodimer. *Antiviral Res.* 177:104760. 10.1016/j.antiviral.2020.104760 32135219

[B71] Zhang K. Donnelly C. J. Haeusler A. R. Grima J. C. Machamer J. B. Steinwald P. (2015). The C9orf72 repeat expansion disrupts nucleocytoplasmic transport. *Nature* 525 56–61. 10.1038/nature14973 26308891 PMC4800742

