# Supplementary material for: Importin-Mediated Pathological Tau Nuclear Translocation Causes Disruption of the Nuclear Lamina, TDP-43 Mislocalization and Cell Death
Source: Front Mol Neurosci. 2022 May 3;15:888420. doi: 10.3389/fnmol.2022.888420 (PMC9113199; doi:10.3389/fnmol.2022.888420)
Supplement: Supplementary file 1 [file Data_Sheet_1.docx]

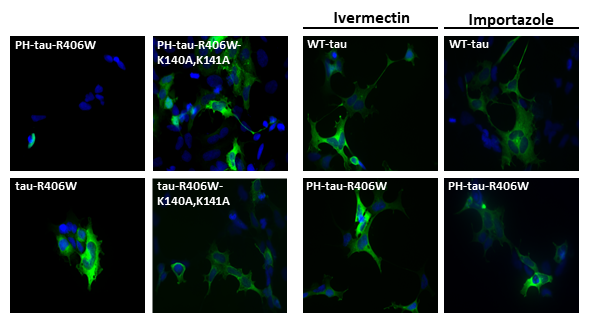


**Figure S1. The R406W mutation does not affect the nuclear import of PH-tau.** HEK-293 cells were transfected with WT-tau, PH-tau-R406W, PH-tau-R406W-K140A,K141A, tau-R406W, or tau-R406W-K140A,K141A. Additionally, cells were incubated with the importin-α inhibitor ivermectin, the importin-β inhibitor importazole, or in drug-free conditions. PH-tau-R406W, but not tau-R406W, translocates into the nucleus (arrow). Interruption of the putative NLS by mutation prevents this translocation. Inhibition of either importin is also sufficient to prevent nuclear translocation of PH-tau. Images are 40X magnification.


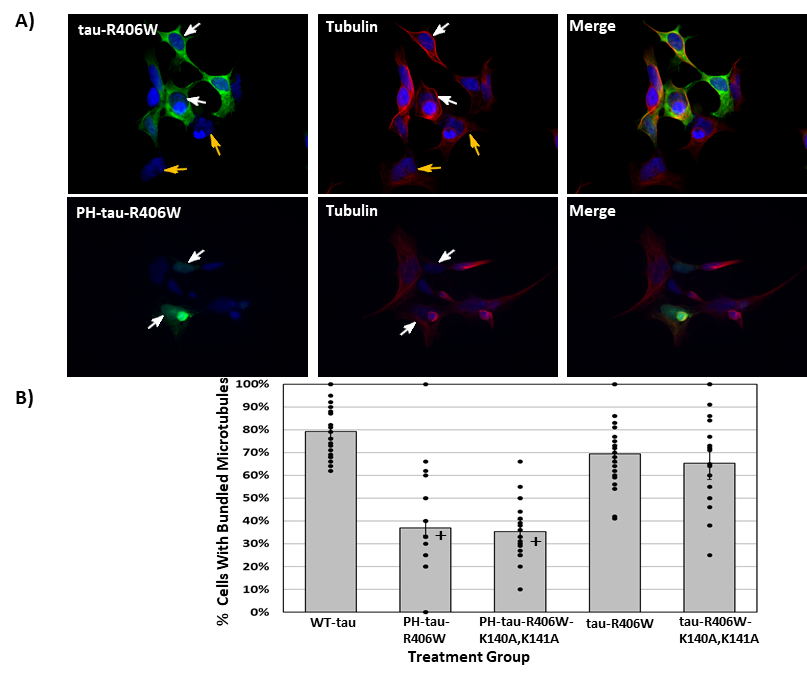


**Figure S2. Microtubule bundling occurs in the presence of tau-R406W but not PH-tau-R406W in a manner independent of its nuclear localization. A)** HEK-293 cells were transfected with WT-tau, PH-tau-R406W, PH-tau-R406W-K140A,K141A, tau-R406W, or tau-R406W-K140A,K141A and immunostained for α-tubulin. Microtubule bundling induced by tau was determined by observation of a ring-like structure around the periphery of the cell. The top set shows the staining in tau-R406W-transfected cells, and the bottom set of images shows the staining in PH-tau-R406W-transfected cells. The images demonstrate the presence of this structure in tau-R406W-positive cells (white arrows, top), but not in tau-negative cells (gold arrows, top and bottom) or PH-tau-R406W-positive cells (white arrows, bottom). Images are 40X magnification. **B)** The number of tau-positive cells in each group that displayed the indicated microtubule structure was counted and expressed as a percentage of all tau-positive cells. The mean percentage across all images (n = 20) was plotted as a bar graph. Error bars indicate SEM. The bars are overlayed with a dot plot of the percentages calculated from individual images (n = 20). Data were analyzed using Welch’s ANOVA, and Games-Howell post-hoc test was used for pairwise comparisons. A **+** indicates a significant difference from WT-tau (p = 0.0017 vs PH-tau-R406W; p = 0.001 vs PH-tau-R406W-K140,K141A), from tau-R406W (p = 0.015 vs PH-tau-R406W; p = 0.03 vs PH-tau-R406W-K140,K141A, and from tau-R406W-K140A,K141A (p = 0.028 vs PH-tau; p = 0.042 vs PH-tau-K140A,K141A).


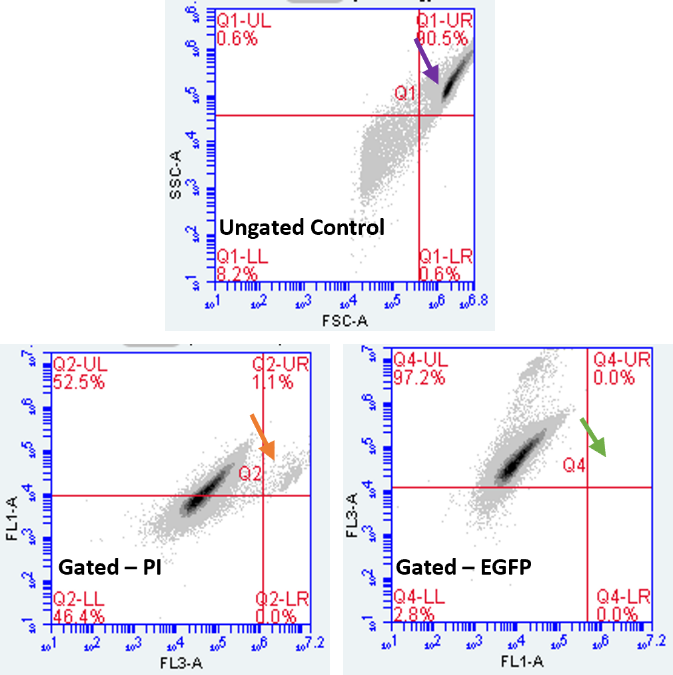


**Figure S3. Gating mechanism for flow cytometry experiments.** Gating was done in two steps. First, we plotted forward scatter vs side scatter to separate cells from debris (see purple arrow in the upper two graphs). Then, this population was plotted with either PI fluorescence (lower left graph) or EGFP fluorescence (lower right graph) on the x-axis. The population of events in the upper right quadrant of these graphs was interpreted as either PI-positive (orange arrow) or EGFP-positive cells (green arrow).


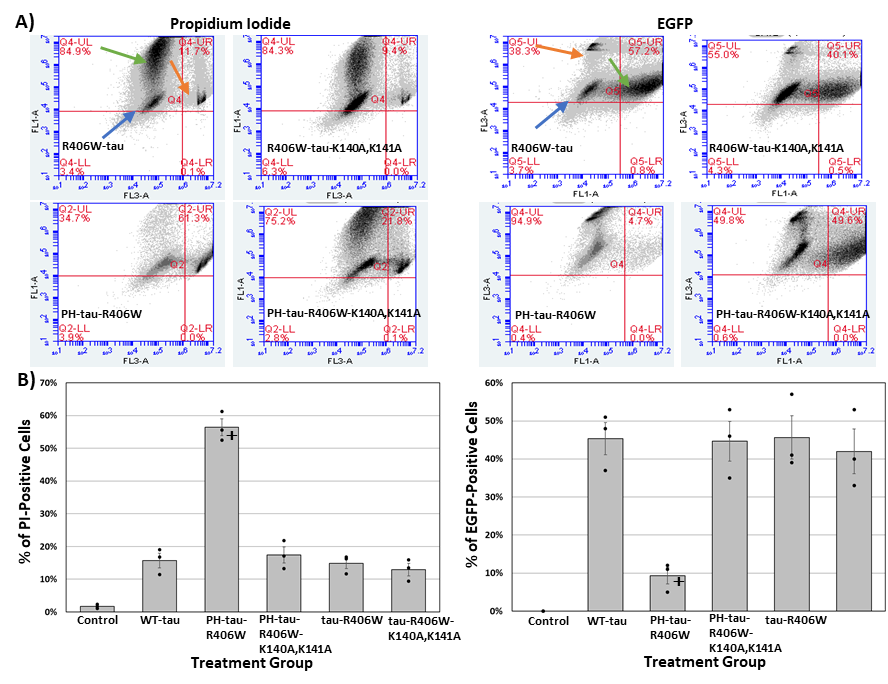


**Figure S4. Translocation of PH-tau-R406W into the nucleus promotes cell death. A)** Graphs indicating the percentage of cells that are PI-positive (left set of four) or EGFP-positive (right set of four) 24 hours after transfection with WT-tau, PH-tau-R406W, PH-tau-R406W-K140A,K141A, tau-R406W, or tau-R406W-K140A,K141A. Orange arrows indicate the population of PI-positive cells, green arrows indicate the population of EGFP-positive cells, and blue arrows indicate the population of cells that is neither EGFP-positive nor PI-positive. **B)** The percentage of events (i.e., cells) was extracted from the upper-right quadrant of each graph and plotted. The left graph shows the percentage of cells that are PI-positive, while the right graph shows the percentage of cells that are EGFP-positive (i.e., tau positive). The average percentage across all 3 replicates of this experiment are plotted as bars, with error bars indicating SEM. The bars are overlayed with a dot plot showing the individual average percentages from each replicate (n = 3). Data were analyzed using Welch’s ANOVA, and Games-Howell post-hoc test was used for pairwise comparisons. The **+** indicates a significant difference in PH-tau-R406W from all other groups, where p < 0.001.


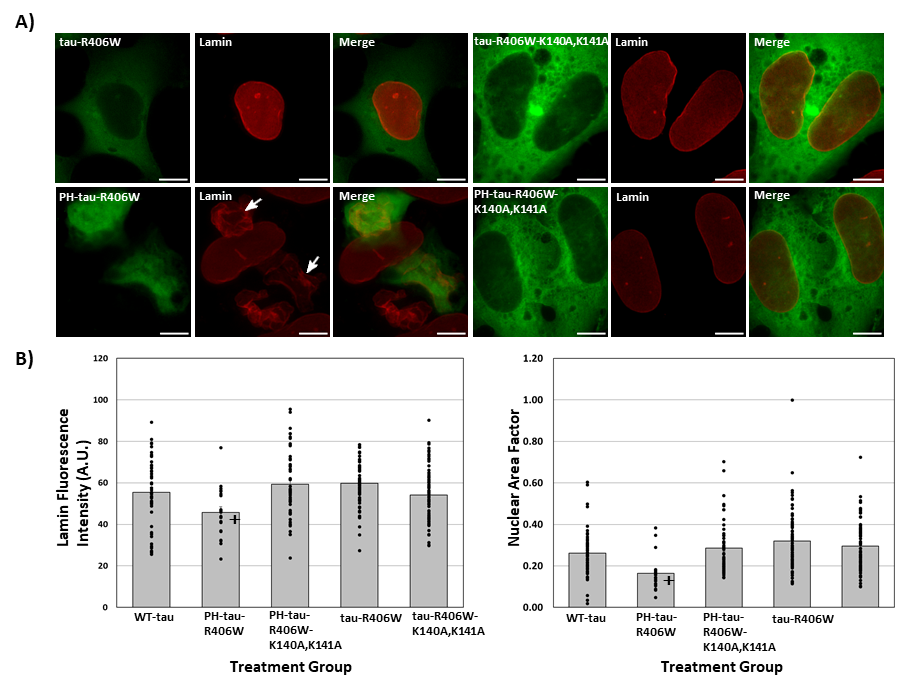


**Figure S5. Nuclear PH-tau-R406W disrupts the nuclear lamina. A)** HEK-293 cells were transfected with WT-tau, PH-tau-R406W, PH-tau-R406W-K140A,K141A, tau-R406W, or tau-R406W-K140A,K141A (green) and then immunostained for lamin-B1 (red). The arrow indicates invagination of the nuclear membrane in a PH-tau-positive cell. Scale bar indicates 15μm. **B)** **Left:** The mean fluorescence intensity of lamin was quantified in individual tau-positive cells using ImageJ. The average fluorescence intensity across all cells (n = 49 for WT-tau, n = 20 for PH-tau-R406W, n = 58 for PH-tau-R406W-K140A,K141A, n = 80 for tau-R406W, n = 80 for tau-R406W-K140A,K141A) was plotted, with error bars showing SEM. The bars are overlayed with a dot plot showing the lamin fluorescence intensity for each cell. Data were analyzed using Welch’s ANOVA, and Games-Howell post-hoc test was used for pairwise comparisons The **+** indicates a significant difference between PH-tau-R406W and PH-tau-R406W-K140,K141 (p = 0.048). No significance was detected in comparing WT-tau and PH-tau. **Right:** ImageJ was used to quantify the area and roundness of each tau-positive nucleus (n = 49 for WT-tau, n = 20 for PH-tau-R406W, n = 58 for PH-tau-R406W-K140A,K141A, n = 80 for tau-R406W, n = 80 for tau-R406W-K140A,K141A), which were then multiplied to obtain the nuclear area factor (NAF). The mean NAF across all cells is plotted, with error bars to show SEM. This plot is overlayed with a dot plot indicating NAF values from individual nuclei. Data were analyzed using Welch’s ANOVA, and Games-Howell post-hoc test was used for pairwise comparisons The **+** indicates a significant difference in PH-tau-R406W compared to WT-tau (p = 0.047) and PH-tau-R406W-K140,K141A, (p = 0.002).


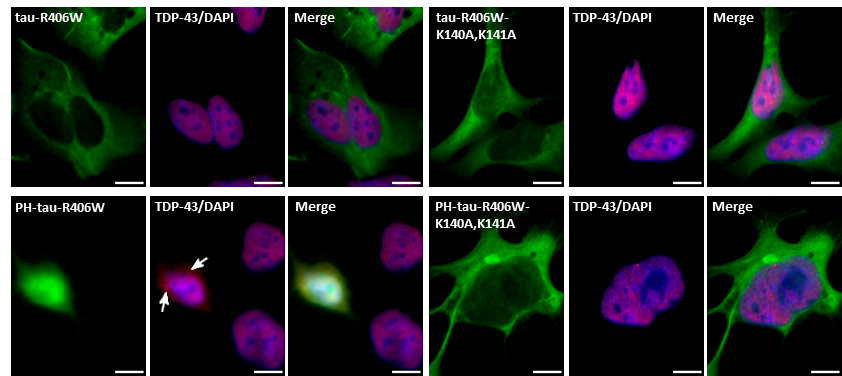


**Figure S6. PH-tau-R406W-positive cells present mislocalization of TDP-43 to the cytoplasm when PH-tau-R406W can enter the nucleus, but not when it is kept in the cytoplasm.** HEK-293 cells were transfected with different forms of tau (green) and immunostained for TDP-43 (red). The localization of TDP-43 was determined by looking for co-localization of the TDP-43 stain and DAPI (blue), which presents as purple. The arrows indicate areas of red staining that does not co-localize with DAPI, indicating mislocalization to the cytoplasm. Scale bar indicates 15μm.


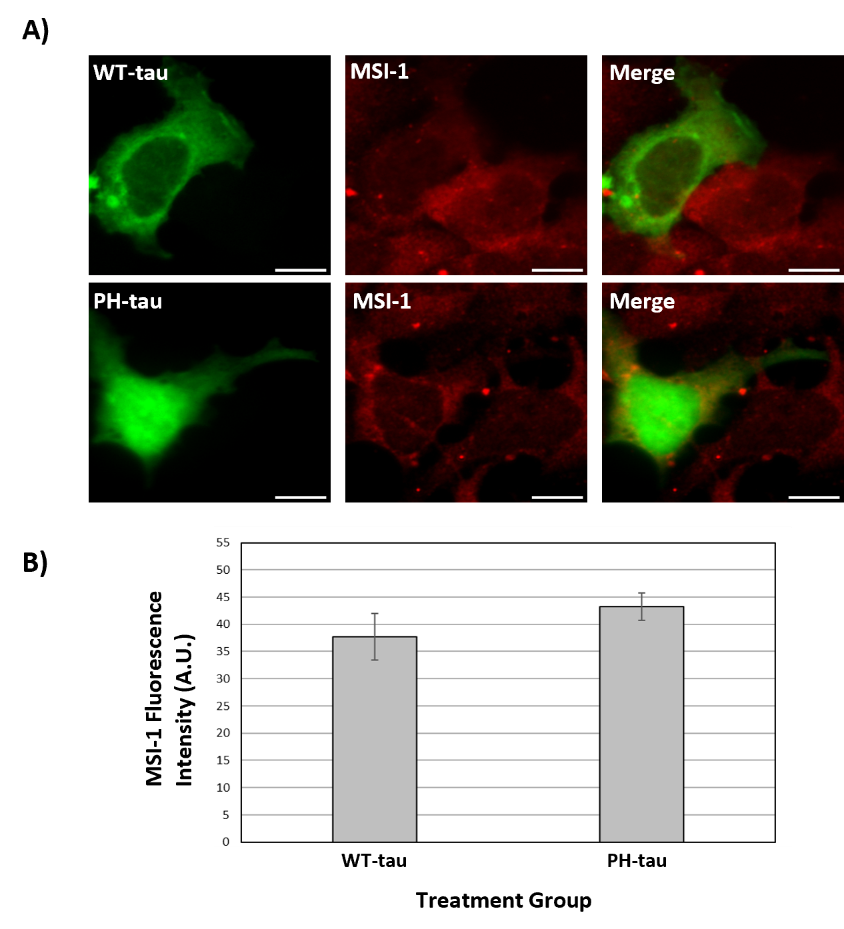


**Figure S7. Expression of PH-tau does not affect the localization or protein levels of Musashi-1.** **A)** HEK-293 cells were transfected with either WT-tau or PH-tau and immunostained for MSI-1 24 hours later. Scale bar indicates 15μm. **B)** Fluorescence intensity of MSI-1 staining was recorded with ImageJ and plotted. Data are mean +/- SEM. No significant differences were detected.


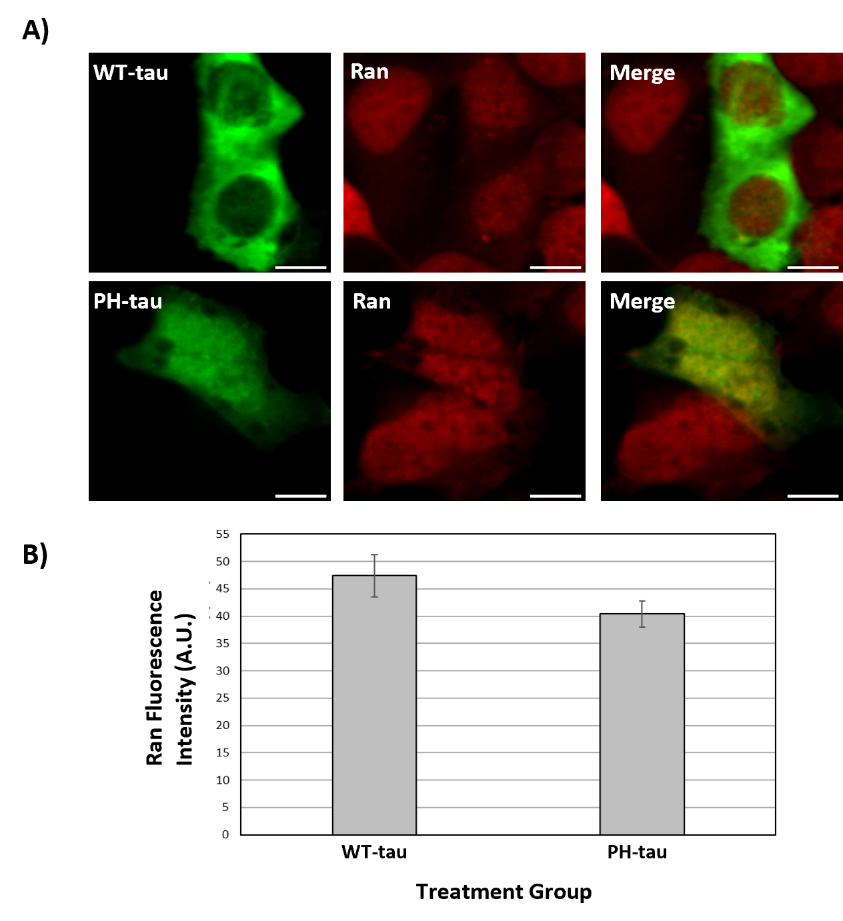


**Figure S8. Expression of PH-tau does not affect the localization or protein levels of Ran.** **A)** HEK-293 cells were transfected with either WT-tau or PH-tau and immunostained for Ran 24 hours later. Scale bar indicates 15μm. **B)** Fluorescence intensity of Ran staining was recorded with ImageJ and plotted. Data are mean +/- SEM. No significant differences were detected.


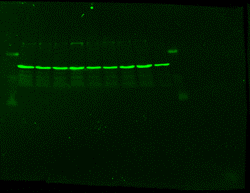

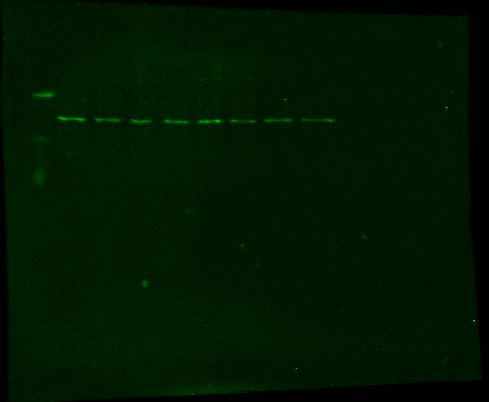


Nucleus – Histone H3

Cytosol -- GAPDH


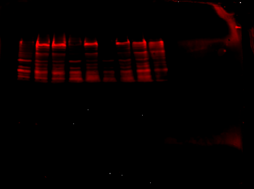

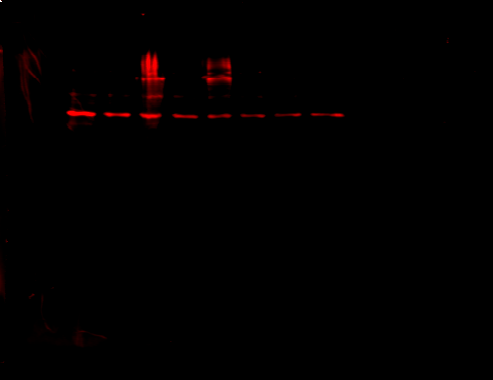


Nucleus -- Tau

Cytosol -- Tau

**Figure S9. Western blots showing tau localization after subcellular fractionation.** Full gels from the analysis in Figure 1C are depicted here. The top row shows the loading controls in green (GAPDH for the cytosol fraction on the left; Histone H3 for the nuclear fraction on the right). The bottom row shows tau in red (cytosol fraction on the left; nuclear fraction on the right).
